# An Essay on Genius and Its Diseases

**Published:** 1819-10

**Authors:** Thomas Middleton Stuart

**Affiliations:** being his Inaugural Thesis for the Degree of Doctor in Medicine, sustained at the University of New-York.


THE LONDON
### Medical and Physical Journal.

4 OF VOL. XLII.] OCTOBER, 1819. [NO. 248.

"For many fortunate discoveries in medicine, and for the detection of numerous "errors, the world is indebted to the rapid circulation of Monthly Journals; "and there never existed any work to which the Faculty in EUROPE and "AMERICA were under deeper obligations than to the Medical and Physical "Journal of London, now forming a long, but an invaluable, series." RUSH.

### Original Communications.

FOR THE LONDON MEDICAL AND PHYSICAL JOURNAL.

### *An Essay on Genius and its Diseases.* By THOMAS MIDDLETON STUART, A.M.; being his Inaugural Thesis for the Degree of Doctor in Medicine, sustained at the University of New-York.

MAN is so constituted, that what is generally esteemed his greatest blessing may become his greatest curse. He has no endowment of body or of mind, which may not prove destructive of his happiness. Strength, beauty, wit, all that can render life desirable or useful, often, through the frailty of human nature, become sources of misery. We may then with propriety, in the following essay, whilst we attempt to speak of genius, treat also of some of the diseases to which its possessors seem peculiarly liable.

Before entering directly upon this subject, we beg leave to premise a few things. The diseases of genius have their seat sometimes in the mind, sometimes in the body, and often affect both body and mind. In treating of these, we may speak of the mind acting upon the body and disordering its functions, and of the body disordering the faculties of the mind: in doing so, many expressions in regard to the mind, through the poverty of language and the imperfection of knowledge, may be used, which are strictly applicable only to what is material. But we utterly disclaim any sentiment favourable to the doctrine of the materiality of the mind, though a Priestley has graced it with his approbation. It would be too distant from our purpose here to attempt a regular refutation of this doctrine, and concerning it we would only observe, that, if the mind be material, it must form a part of the body or the whole body; in other words, every part of the body together must form the mind, or some particular part must form it,—as the brain, or heart, &c.; for, if this be not allowed, the immateriality of the mind must be allowed. The first supposition, viz. that every part of the body together forms the mind, needs no refutation; the second, that some particular part forms it, has more plausibility, but as little truth. We will only notice what Priestley says in favour of the brain and mind being one and the same; for, if it were reasonable to fix on any part of the body as the mind, that part ought to be the brain. Priestley is persuaded of the truth of his doctrine, "because," as he says, "as far as we can judge, the faculty of thinking, and a certain state of the brain, always accompany and correspond to one another." And again, "there is no instance of any man retaining the faculty of thinking, when his brain was destroyed. Moreover, as the faculty of thinking in general ripens and comes to maturity with the body, it is also observed to decay with it; and, if in some cases the mental faculties continue vigorous when the body in general is enfeebled, it is evidently because, in those particular cases, the brain is not much affected by the general cause of weakness. Likewise, as the mind is affected in consequence of the affections of the body and brain, so the body is liable to be reciprocally affected by the affections of the mind, as is evident in the visible effects of all strong passions. These are certain irrefragable arguments, that it is no other than one and the same thing that is subject to these affections."

In answer to these irrefragable arguments, we oppose *facts* to *assertions*. "The history of dissections proves that the texture of every part of the brain may be morbidly altered from its natural state, and yet all the faculties of the mind remain entire." To this the writings of Morgagni, Bonetus, and Haller, bear testimony.* "Portions of the brain have been forcibly detached; excavations have been formed in it by abscesses; fungous tumors have arisen from its surface; all its arteries have been ossified; its coats have been variously diseased; the interior part of the cerebrum and of the cerebellum, the basis of the cerebrum, the pituitary gland, the pineal gland, the plexus choroides, have all been found exhibiting morbid changes of structure, in people who were in full possession of their internal senses."† Instances occurred in Dr. Hunter's dissecting-room, where the brain was found almost entirely converted into pus, and yet the persons in whom the brain was thus found diseased had suffered no mental derangement.‡ How then stands the assertion, that "there is no instance of any man retaining the faculty of thinking, when his brain was destroyed?" When the body is affected with palsy, and yet the faculties of the mind

* See Morgagni *de Causis et Sedibus*, Epist. xi. xv. xx. and the first volume of the *Memoirs of the Literary and Philosophical Society of Manchester*.

† Crichton on *Mental Derangement*, vol. i. p. 241.

‡ Fordyce on *Fevers*, vol. ii. p. 102.

remain entire, and when, on examination after death, disorder of the brain is discovered to have been the cause of the palsy, shall we say, that the faculties of the mind were not injured, because the *brain* was not much affected by the general cause of weakness? There is scarcely a disease that can affect the body, by which the brain, from its extensive connexions, must not be more or less affected: but who, that has walked much in the paths of sickness and death, has not occasionally seen, where the powers of life were fast sinking and just ready to fail, the mind acquiring fresh strength, as if already partly relieved from its clay? While many of the phenomena of disease show that there is generally an intimate connexion between a healthy state of the brain and a proper exercise of the mind, the facts we have adduced, prove that the brain and mind are not "one and the same," and that occasionally the mind acts independently of the brain. In proof that the faculties of the mind do not necessarily decay with the body, we might mention the venerable names of Plato, Isocrates, Newton, Johnson, Burnet, Burke, and many others, who exhibited to the world the splendid and interesting spectacle of bodies shattered by age and worn-out by disease, with minds retaining all their greatness, and uninjured amidst the ruins that surrounded them. We ask those from whom we may differ, if any instance can be adduced, in which the faculties of the mind have decayed, while the mind has been cultivated with the same assiduity in old age as formerly? In old age stimulants to exertion are wanting, therefore the mental faculties are too often allowed to remain inactive; then indeed they do decay; but we conclude with Cicero, "manent ingenia senibus, modo permaneat studium et industria." It is time to turn to our proper subject.

An enquiry into the nature of genius is interesting, inasmuch as it is an enquiry concerning that which is productive of the greatest benefit, as well as of the greatest ill, to mankind. Its effects are felt in every department of life, whether it reside with the rustic, making him the counsellor of his hamlet; with the philosopher, placing him in the van of the march of science; or with the statesman, constituting him the shield of his country; whether it glow upon the lips of oratory, or encircle with its halo the visions of poetry. They are exhibited in the prosperity of nations, and in their ruin. It clothes its possessor with that mental majesty, which extorts from envy obedience and admiration,—to which the weak look for protection, and the brave for guidance.

Thus is genius exhibited in its effects:—But what is it? Borrowing partly from Dr. Johnson, we define it "a mind of superior general powers." We cannot suppose that genius consists in the superiority of any one power of the mind, or in the combination of any particular powers; and think, that a brief survey of the subject will show, that, as the beauty of a palace does not consist in the elegance of one pillar, but in the proportions and skill exhibited in all its parts, so genius does not depend on the excellence of any one power of the mind, but on the vigour and combination of all its powers.

Invention is the criterion of genius. This seems to be allowed by common consent; for all whose characters are established in the world as men of genius, are those who have invented something unknown before, or invented improvements in what was already known. Homer, without a model, constructed one of the noblest poems the world possesses; Socrates invented moral philosophy; the names of Newton and Franklin are illustrious by their discoveries in physics; the memory of Fulton is immortal by his application of the powers of steam to the purposes of navigation; and Sir Humphry Davy has placed himself in the foremost rank among philosophers, by his improvements in chemical science. That invention is the criterion of genius being granted, it is incumbent on us to show how the vigorous exercise of all the powers of the mind are necessary to the act of invention.

The intellectual powers we will consider, adopting Girard's division, as consisting of sense, memory, imagination, and judgment. "Sense perceives those objects which are really existent, and actually exhibited to the mind." Memory revises "past perceptions, with a view to experience of them and to their reality. Imagination considers the notion or thought present in the mind simply as it is in itself, without any view to real existence or past experience,"* and, by association, leads from the idea present in the mind to others connected with it. Judgment compares ideas presented to the mind, discovers their relations, and draws conclusions concerning them.

In the act of invention, it is imagination which ranges through the regions of thought, conducted by association from one idea presented to the mind, to the various others connected with it, separating notions or parts of notions from those with which they were formerly associated;—again combining these into new forms, with a semblance of creative power;—like the bee, collecting honey from every flower on which it rests, however various be the tint, or dissimilar the general qualities. But, to possess that superiority which will lead to invention, imagination must be attended by *enthusiastic activity*, which constantly stimulates it to new excursions, quickly presents it with new views of objects, rapidly separates old connexions of ideas, and as rapidly re-combines them into every form of which they are

* See Girard on *Genius*, and Beattie on the *Imagination*.

susceptible, which so absorbs it in the contemplation of its object as to prevent any interruption of attention, and so fires it, in its pursuit, as to render it careless of every fatigue and labour it may undergo. It was this enthusiastic activity with which Archimedes merged the noise of battle and the terrors of death in his mathematical speculations;—which trimmed the midnight lamp, and dug the early grave, of Henry Kirke White. Without activity of imagination, the mind soon tires in the investigation of a subject; and, however excellent and new may be the associations which are formed, their excellence and novelty rarely compensate for the tardiness with which they arise; and without enthusiasm, as the exercise of imagination, however active, is still attended by much fatiguing exertion, the mind will soon relax in its endeavours, and consider that unattainable which merely has not yet been attained. It is the observation of common life, that enthusiasm, in the pursuit of any object, is one of the surest tokens of ultimate success.

Imagination, however active, can do little towards invention, unless it be comprehensive. It may elicit a spark which will dazzle for a moment, but can never produce that steady lustre which leads to useful results. It is comprehensiveness of imagination, which gives to the productions of genius, that richness which appears to be formed by a happy selection from a vast collection of materials submitted to its choice;—which unfolds to the mind the view of its subject in all its parts, and in all its connexions;—which enables the associating principle to traverse every region, and explore every recess, in quest of its treasures; —and to pass from one idea to another, and through all their modifications, with seemingly unlimited bounds. It was by a comprehensive imagination, that the great poets and philosophers of ancient and modern times have collected in their works materials from every region of nature and art; and have seemed to hold within their reach the universe of ideas, from which to draw all that was conducive to their purpose.

In a work of genius, imagination, active and comprehensive as we have described, surrounds itself with a lustre, which, from hasty observation, conceals the operation of other powers. But, for the perfection of invention, the aid of other agents is essentially necessary. Indeed, without sense and memory,* imagination itself cannot act; for it must be from some one object perceived by sense that association begins, and it must be by the help afforded by memory, that it continues its exercise. Sense is the fountain from which association springs; memory,

* "Memory," says the author of the Literary Character, "is the foundation of genius; for this faculty, with men of genius, is associated with imagination and passion: it is a chronology, not merely of events, but of emotions."—*Lit. Character*, p. 127.

the tributary streams by which it is enabled to roll on its fertilizing course. The objects of imagination are all confined within the limits of acquired knowledge: these it may variously modify, but cannot go beyond them. It is impossible for it to act on those things with which it has no acquaintance by experience or information. When the fervid imagination of Milton mounts to heaven,—is conversant with cherubim,—is immersed in the splendour of the Supreme,—and seems to leave, far below, earth and its grossness, still we find it adoring heaven with the materials of this world;—giving to archangels human forms,—and, by what appears glorious among men, describing the glories of the Creator of worlds.

By a hasty sketch, we have shown how far imagination, with the aid of sense and memory, can proceed in the act of invention. Imagination collects the materials, but the choice and arrangement is the work of judgment;—which compares ideas presented to the mind, discovers their relations, and draws conclusions concerning them. Not only is judgment called into exercise when the collected treasures of imagination are presented to the mind, but the imagination of genius, even in its most free and independent excursions, must still be attended by that power, which detaches it from improper and useless associations, directs it to the track most conducive to the end in view, and calls it off from chasing the cheating shadow of a cloud; and, when imagination has collected its various materials, judgment is the spirit moving over the rich but confused mass, educing order and usefulness. A writer of much imagination, not tempered by judgment, will produce extravagance and folly; but, where sound judgment is connected with an active, comprehensive imagination, the happiest results are to be anticipated,—the production not only of what is new, but what is congruous, useful, great; for there is not a flower that strews the path of genius, nor a column in the proud structure that it rears, in which the influence of judgment is not concerned. The continual influence of judgment on imagination, does by no means injure the exercise of the latter, only renders it more efficient: the ship does not less feel the useful influence of the breeze, on account of the directing helm and rudder.

We thus conclude, that genius consists in a combination of all the powers of the mind. It is this combination which gives intellectual strength, which enables one mind to influence thousands. In proportion as these powers are fully possessed, and properly combined, so far does genius exist.

Whether superiority of genius be natural or acquired, we will not stop here to examine, nor pretend positively to determine. The power of education daily shows itself to be so great, that we know not how to limit its effects; while the records of men of genius exhibit such peculiarities of character, as almost persuade that

> "from heaven descends

> The flame of genius to the *chosen breast!*"

One thing must be allowed,—that the circumstances of life, and especially of early life, tend much to determine the characters of men. But the same circumstances produce different effects on different minds: a peculiar concurrence of circumstances, whilst it elevates one mind, may degrade another. We often see men labouring under circumstances apparently the most adverse, and still exhibiting splendid marks of genius; but it is probable, that what appear adverse, are the very causes of the display of genius; and that the same persons, in situations seemingly much better adapted for the exercise of talent, would fall into obscurity. Had Newton been a printer's boy, or Franklin been educated at an university, possibly neither of their names would have been rendered immortal by their discoveries in natural philosophy.

According to our description, there is a complex operation of the mind in every exertion of genius; it may therefore readily be supposed to be liable to disorder. This may arise, 1st, from inactivity; 2d, from imperfection; 3d, from artificial means of excitement; 4th, from excessive exercise.

1st. In some respects, the mind appears to be governed by laws analogous to those which govern the body; for, as continued inaction deprives the body of the power of moving, so *inactivity of genius* deprives it gradually of the power of acting. To this in a great measure is to be ascribed that want of mental vigour which so often attends declining age. At this period, fame ceases her allurements,—ambition begins to lose its power,—life has little more to promise,—the animal spirits are languid,—infirmity unfits the body for the support of the exercise of mind,—and the individual gradually and unconsciously remits that exercise, and at last falls into dotage. But not only in old age does inactivity show its deleterious effects on genius. We often see or hear of men, who in early life gave the fairest promise of future eminence, who, when they have reached their prime, not merely have made no progress in mental improvement, but have gone backwards; and, together with the loss of acquired knowledge, have lost the thirst for it; exhibiting the pitiable sight of the waste of talents and the wreck of mind. This may sometimes be occasioned by their having been placed in circumstances unfavourable to their peculiar turn of mind:

> "many a soul sublime
> Has felt the influence of malignant star."

But often another cause is very evident. Elated with the praise bestowed upon their early talents, they conceive that, without regular exertion, which is the pabulum of the mind, they can, by their superior powers, attain any object: depending on this, they allow inactivity, like a canker, to eat out the soul and strength of genius.

2d. Although we have described genius as formed of a proper combination of all the powers of the mind, yet, as the mind of man is never perfect, we are compelled to denominate that genius, in which however there are some imperfections, some disproportion in the parts which compose it. Its occasionally great achievements, when circumstances have conspired to repress that power whose action is inordinate, or stimulate that which is languid, establish its claim to this distinction. These imperfections are sometimes causes of disease. We may consider them as circumstantial or essential. Circumstantial imperfections, are those arising from a peculiar constitution of the man of genius, but not implying any deficiency or disproportion in the powers of mind which constitute genius. Essential imperfections, are those which imply some deficiency or disproportion in those powers, as when the action of judgment is not able always to control that of imagination.

Among the circumstantial imperfections of genius, we notice *timidity* and *want of perseverance*. In attempting any work, genius will generally have an extensive view of the difficulties to be encountered: it will measure the region to be explored, will mark what rocks are to be levelled, what valleys are to be raised, what wilds are to be explored. After such a view, constitutional timidity, alarmed by the obstacles which present themselves, too often dissuades genius from attempting what it is well able to perform. As timidity oft prevents the attempt, want of perseverance oft prevents the accomplishment of great undertakings. Both are productive of very deleterious consequences. Timidity, by infusing into the mind capable of forming great designs groundless fear of inability for the accomplishment, throws it into a state nearly allied to despair; genius, parched with thirst, sees the cooling stream roll before it, without the hope of ever tasting its refreshment; and, shackled by want of perseverance, must be the prey of grief and chagrin, by seeing that honourable distinction gained by others which it failed to secure when almost within its grasp. That the feelings of a man of genius, in such circumstances, will be exquisitely afflicting, the history of genius gives strong ground for concluding. Honourable fame is dear to genius; and few will censure the mournful exclamation of Kirke White, when he saw the grave opening to receive his body, and as he thought his dearly-earned reputation, from the world: "Fifty years hence, and who will hear of Henry!" We find those, who had no cause to reproach themselves with want of exertion, and who had accomplished great designs, yet sinking into despondency on seeing themselves surpassed by others. He is rare who can comfort himself with the reflection, that "Sparta has many a worthier son than he." "When some of Morillo's paintings were shown to Castillo, the great painter of Seville, he stood in meek astonishment before them; and, when he recovered his voice, turning away, he exclaimed with a sigh, '*Yà murio Castillo!*'—Castillo is no more! Returning home, the stricken genius relinquished his pencil, and pined away in hopelessness." If such be the anguish of those who, though excelled, were true to their inspiration, what must be his sorrow who sees that crown grace the head of another, which, but for want of exertion or perseverance, might have bloomed upon his own! Settled melancholy must often be the effect of such sorrow. Observation shows the effects of grief to be debilitating to the whole corporeal system: hence the torpidity, the pale countenance, the cold extremities, the disinclination for muscular motion or mental exertion, which attend its victims. From the diminished circulation in the extremities and surface, arises the painful sense of fullness which is attendant on deep sorrow; as the larger vessels of the body must thus necessarily be over-distended with blood. That there is an intimate connexion between the skin and stomach, is a principle well established in medicine: the action of the vessels of the skin being diminished, the stomach is affected, probably through the connexion of nerves; while, at the same time, the internal vessels, receiving an inordinate quantity of blood, expose that organ to still greater disorder: and hence arises that acute pain in the pit of the stomach, which grief so often produces, especially in nervous temperaments. That the over-distension of the internal vessels is concerned in this affection, receives much probability from the observation of Crichton,* who says that he has seen this kind of gastrodynia, in two instances, followed by hæmorrhage from the stomach, lungs, and uterus. The same causes will produce plethora of the vessels of the liver, and consequent disease of that viscus. These disorders, thus produced by grief, re-act upon it, and aggravate that dejection of mind from which they at first originated; for, produced by any cause, depression of mind is one of their invariable consequences. When added to original grief, they act with a force which few minds can withstand. Deep sorrow will take so firm a hold upon its victim, as to preclude from his thoughts every subject not connected with his unhappiness. Life has its shade and its light; but his mental vision is active only in its darkness: he becomes so enamoured

* Crichton on *Mental Derangement*, vol. ii. p. 190.

with misery, as to flee from the comforts of friendship, and the calls of business or pleasure, that he may indulge his gloomy meditations: the sand of life ebbs in its glass, the flame of genius quivers in its socket,—he dies! the pity, if not the contempt, of his acquaintance! This is the melancholy effect which may follow timidity in not attempting, and want of perseverance in not executing, the designs of genius.

The only *essential* imperfection of genius which we will now cursorily notice, is that in which the imagination is disproportionate, in its action, to the judgment. In our description of genius, we mentioned that the speculations of one with a great disproportion of this kind in the powers of the mind, would end in folly;—it has often ended in insanity, as the records of every lunatic asylum testify:* but, even when existing in a smaller degree, it is often productive of baneful consequences. While it constantly exposes its possessor to errors and imprudencies in conduct, which, in a well-proportioned mind, judgment would have prevented, it makes him liable also to the most cruel disappointments. By a morbidly-active imagination, hypotheses, which have but the semblance of probability, are embraced as truths; it sees a friend in every smile, and hears the promise of success in every whisper of hope; it never supposes that the day which is bright at the dawn, can blacken with clouds; and concludes, that the attainment of its object of pursuit will be as easy as its desirés are ardent. Thus acting beyond the control of judgment, if that power is not altogether dispossessed of its seat in the mind, the consequence, at least, must be disappointment. The best-concerted schemes are often thwarted, well-founded hope is often deceived; then how frequent must be his failure and dissatisfaction, who begins to build without counting the cost, who has never considered that deception smiles, or that friends can betray,—that hope can be illusive, or merit envied. Chagrin often occurring, as it will, in the paths of such an one, is apt to drive him to a contrary extreme: some having deceived, he supposes himself the object of the deception of all; the world seems his enemy, every misfortune is exaggerated; falsehood having betrayed him, he now suspects every kindness, and mistrusts every promise; having failed of obtaining his objects by not employing proper means, those objects, he conceives, by some malignant fatality, are placed beyond his reach; disappointment preys upon him, and too often produces that gloomy melancholy, with its direful consequences on mind and body, which we have already described.

3d. In a state exposed to vicissitude and vexation, encumbered with a body liable to lassitude, sickness, and pain, genius

* See Rush on the *Mind*, p. 38.

4

is often chained in its exertions; no wonder that it hails with pleasure what can set it free: shut out from the paradise of its meditations, it readily yields to any influence that can open the gates of lost happiness. With the powers of his mind languid or ruffled, the man of genius, to restore their activity and soothe their commotion, too often resorts to artificial means: alcohol and opium are both subservient to his purpose. We will now only notice opium, as thus used, and some of its hurtful effects. The effects of this drug, in exciting the mind to action, is peculiarly enticing, especially to those who revel in the regions of fancy: under its influence, the storm of passions is hushed to a calm, the clouds of despondency dissolve away, and the mental faculties seem to acquire new strength and splendour. But this state of enjoyment does not last long; the charm is soon broken, and more than in proportion to former elation and enjoyment is the depression and misery that follow. This is according to the experience of all who have observed the effects of opium on themselves or others. The depressing consequences of each dose taken is a new inducement to repeat it, and at the repetition of each dose habit calls for a larger portion. The deleterious effects of indulgence in it, on mind and body, are various. The effects on the body are, dyspepsia, and all the consequences of diminished secretions. "Probat, ab usu hujus remedii (opii) diuturniore, organa chylopoiesi et sanguificationi inservientia, adeo debilitari posse, ut officiis suis imparia reddantur." "Experimenta Alstoni opium circuitum in vasis minimis, priusquam in majoribus, cohibere probant." "Secretiones cohibet." "Alvum constipat."—Bard *de viribus opii.*

Under the effects of opium, the body is rendered less susceptible of external impressions, on account of the diminished circulation in the smaller vessels; and this, perhaps, conduces to produce increased vigour of mind, by preventing interruption of thought. When those effects have ceased, the circulation, returning to the smaller vessels, will render the nerves connected with them morbidly sensible, and hence more liable to violent impressions. We now perceive how the continued use of opium may produce disorder of mind: the viscera of the body are diseased, which, as we before observed, produces a depressing affection of the mind; the nervous system is irritable, and the mind often sympathizes in its irritations; the mind is often at the height of joy, or in the depth of misery, through the excitement of opium, or the want of it; and Rush observes, that frequent and rapid transitions from one subject to another, is a cause of intellectual derangement. These combined consequences, if they do not produce mania, often produce that modification of it generally called hypochondriasis, or, as we think, more properly styled by Rush, tristimania.

Perhaps it is scarcely affirming too much when we say, that, after a person has long indulged in the use of opium, whenever he is not under its inebriating influence, (that is, whenever he is himself,) his mind is more or less deranged; for it is then deprived of its powers of action, and sees objects in a false light.

4th. We will next attend to the diseases of genius arising from *excessive exercise.* The mind, as well as the body, is rendered by proper exercise more strong and healthy; but is liable to much harm from exertion very violent or too long continued. As the mind is affected by fatigue of body, so is the body by fatigue of mind. Men of genius, who, aided by industry, are enabled to make deep researches, and take comprehensive views in science, to range among objects which inferior minds dare not attempt, are too apt to exert the mind to excess. Bodily feeling is lost in the ardour and intensity of intellectual exertion, and it is not till the mind has in some measure attained its end, and has begun to rest from its labour, that the hurtful effects on the body are perceived: then is felt langour, depression, anxiety, and restlessness, in proportion as the mind has been laboriously employed. Excessive exertion of mind, long continued or often repeated, soon affects the organs of digestion; and hence frequently originates that distressing mental complaint, well denominated tristimania.

In any great exertion of the mental faculties, there appears to be a considerable flow of blood to the head, occasioning there some degree of congestion;* as is evident from the head-ach, vertigo, redness of the face and eyes, which occur in debilitated persons from a very slight exertion of mind, and in healthy persons, when the exertion has been uncommonly great. This flow of blood to the head, acting as a preternatural stimulus to the blood-vessels of the brain, is followed by indirect debility of the brain. Through the connexion that exists between the different parts of the body, its other organs will sympathize in this debility; which also is partly the cause of that mental depression and anxiety which attends disease of the body, from too great exertion of mind; for, as the perceptions of external objects are conveyed to the mind through the medium of the nerves and brain, these being in a disordered state, must generally affect the functions of the mind.

Moreover, debility of body from mental exertion seems also to arise from this, that, while there is a preternatural flow of blood to the brain, the secretions of the body in general are lessened; the organs of digestion and chylification are deprived of their proper action, as the diseases of studious men plainly

* Crichton on *Mental Derangement*, vol. ii. p. 29.

evince; the intestinal canal loses its due irritability; for it is found that cathartic medicines are much slower in their operation on one who is engaged in study, than on another whose mind is unoccupied.

The body is also weakened by loss of sleep, which is often the effect of intense application of mind to any subject; for it is found that, when the attention has been very much engaged on any particular object, the associations thereby excited continue to act long after all voluntary exertions of attention have ceased: every one must have observed this. Boerhaave mentions, that, having been exercised with intense thought during a whole night on a serious subject, he did not sleep for two weeks, and during that time was perfectly indifferent to everything around him. Zimmerman, in his work on Experience in Physic, mentions the case of a young man, which strongly exemplifies the hurtful effects of intense study. This person engaged very ardently in the study of metaphysics; and, after combating, by increased exertion, an inertness of mind which he perceived coming upon him, lost at last the exercise of every mental faculty, while his bodily health was much injured: after some time his body recovered its wonted health, but his mental disease continued for a year: "without being deaf, he seemed not to hear; without being blind, he appeared not to see; without being dumb, he did not speak." He became afterwards an eminent philosopher. Many instances might be adduced, in which intellectual exertion has proved destructive of life. Too frequent is the melancholy record of genius snatched from the hopes of friends and the world, when its budding flower just began to give promise of the rich fruit that would be produced. Were bodily disease the only evil resulting from over-exercise of genius, it might admit of question, whether, as life is short and uncertain at best, it were not better, in order to make greater improvement in a shorter time, even like Kirke White —to die: but sad experience shows, that not only the body, but the mind also, is diseased by its own exertions.

One disease of the mind thus arising, and intimately connected with the corporeal affections already noticed, is tristimania. This disease of the mind often, perhaps it is better to say generally, arises from disease of the digestive organs. Why disorder of those organs so generally produces this distressing complaint, may be explained in the following manner: The nerves of the stomach, and parts connected with it, are subject to impressions of a peculiar kind, but which are never so powerful as to attract any particular attention of the mind to them; but, when these organs are diseased, unaccustomed impressions are made upon their nerves, both by the disease of the parts themselves, and by the aliment taken into the stomach not having undergone the proper process of digestion. Moreover, while new impressions are thus made on the nerves of the digestive organs, these new impressions are transmitted to the brain with more force than the old ones were wont to be, on account of the excitability produced in the brain, from the preternatural stimulus applied to it, by the inordinate flow of blood thereto, which we before remarked as a consequence of intense study. The mind, being acted upon by these impressions, which are new, from the unnatural state of the digestive organs and of the stimuli applied to them, which are strong, from the peculiarly irritable condition of the brain, and which are often painful, is alarmed, and, not guided by experience in referring them to their proper cause, is often led to assign the most absurd. Hence the numerous anecdotes which are related of persons supposing they had living animals within them; that they had been changed into wood, glass, &c.; that they had been poisoned, or changed into animals of a different species. We will not intrude a repetition of these. From no disease does man suffer more than from that whose cause we have just endeavoured to explain. During its paroxysms, (for it is observed to have paroxysms,*) all the faculties of the mind cease their useful action, and are exercised with the most severe suffering: genius loses its pre-eminence; and the torment of the patient is such, that he often wishes for, and often seeks, death as a relief. Tristimania, as well as that other form of mental disease which we shall presently mention, often terminates in general intellectual derangement, which perhaps always precedes suicide.

The next disease which we notice, as arising from excessive exercise of the mental faculties, is that which is caused by intense study upon a particular subject, the full comprehension of which may be within the scope of human intellect, but is oftener beyond it. Mental perceptions appear to have corresponding sensorial impressions, which are transmitted to the extremities of different nerves with a force proportioned to the vividness of the mental perceptions. When the extremities of nerves are strongly impressed, we are liable to ascribe the impression to external objects, through the dictates of experience; so a person of full habit stooping down, and thereby occasioning some congestion of blood in the vessels of the head, which produces an unusual impression upon the optic nerve, is led to believe the spots, &c. which he sees, to be objects without him, until better informed. Now, the brain being in a state of excitement from intense study, and receiving repeated impressions from one particular set of notions indulged by the mind, it is easy to conceive that these impressions may, at last, acquire a

* See Rush on the *Mind*, p. 38.

vividness unnatural to any but those received from external objects; and hence the subject of such impressions is led to believe in the real existence of that which has a being only in his own thoughts. Particularly is this apt to be the case, when the subject of study is one beyond the reach of human capacity. The exertion of the mind must be in proportion to the difficulty of the subject; but, when that is incomprehensible, the mind, violently exerted, enjoys little rest from that exertion: once ardently engaged in the pursuit of any object, it rarely ceases pursuit till in some measure it has attained its end; when that end is unattainable, its strenuous, constant, and vain endeavours, must produce a state of brain peculiarly liable to mental illusion. Crichton, (vol. ii. p. 65,) after having adduced a number of examples, illustrative of delirium produced by intense application to subjects of study which give an extraordinary degree of force to imagination, observes, "the examples which have been brought forward are surely quite sufficient to prove the melancholy truth of the hypothesis laid down, that representations of the mind, when frequently renewed by acts of the imagination, at last acquire a degree of vividness which surpasses those derived from external objects; and, as the principal quality of a mental perception or representation, which makes us believe in the reality of the object or objects which it represents, is the clearness of its parts, it is not wonderful that men of genius, who often confine their attention to one branch of study, should be more exposed to such illusions than any other class of people." Hence, we find persons who have engaged in the study of the Trinity, with a desire of comprehending the manner of the union of three persons in one Godhead, (a doctrine which, though written with a sun-beam in Scripture, still we cannot hope to comprehend,) have frequently supposed themselves to be one of the Trinity. The intense study of other subjects connected with the Christian religion, which are given us as objects of faith, which have testimony of their divine origin, but which, while the mind is veiled by its clay, must remain mysterious, have produced a similar effect. More than one person, by vainly endeavouring to ascertain the precise time when the millenium should arrive, have had all the faculties of mind so absorbed in its contemplation, as to be applied to nothing else; and have had those faculties so deranged, as, with a presumption of prescience, to fix the date of its arrival, or declare that it was already at hand. The Rev. John Mason, of Water Stratford, near Buckingham, England, died fully persuaded that, as the Elias, he had a divine mission from Jesus, to announce the approach of the millenium, which was to begin in his neighbourhood. Professor Gruner, of Jena, mentions the case of a student of theology, at Leipsic, who, from intense study of the Revelations of St. John, had his intellects so disordered, as to consider himself divinely inspired, and who at last became so deranged as to murder his father. From the same source are to be derived the dreams of Baron Swedenborg, and of the amiable St. Theresa of Spain. The Christian religion stands unreproached by any of these consequences: it never produced them. They were the result of an attempt to learn that which it never taught. Its doctrines, sincerely and humbly received, are the surest guards against mental disease: the subject of them is enabled to stand unchanged amid the changes of the world,—amid its convulsions, unshaken; to bear prosperity with equanimity, and adversity with fortitude; to bound joy within its due limits, and to restrain grief from excess; to fix hope on its proper object, and to lose fear in the confidence of mercy.

In the study of all arts and sciences, the species of mental disease of which we have spoken, has been produced by similar causes,—by attempts to discover that which is not discoverable, to know what cannot be known. Perpetual motion, the quadrature of the circle, and the philosopher's stone, have destroyed more than one mind.

For interesting cases, illustrative of tristimania, and of the last-mentioned mental disease, which falls under Rush's description of amenomania, we refer to the writings of that great man, and to Crichton on Mental Derangement.

In closing our subject, we would mention a few means which appear useful in preventing diseases of genius. As in the body, so in the mind, different persons are so differently constituted, that what would be excessive exercise in one would be moderate in another: we cannot establish any standard in this respect. It is evident that, to prevent disease from exertion of genius, we must prevent too great exertion. Our feelings must be our guide in this; and a sense of bodily fatigue, weariness, a kind of fullness and tension of the forehead, often producing severe head-ach, are faithful monitors that the mind needs rest and relaxation. When Buffon was absorbed on a subject which presented great objections to his opinions, he felt his head burn, and his countenance became flushed; and this was a warning to him to suspend his attention. A person deeply engaged in study may not attend to these feelings; but this inattention arises from no idea of danger having been associated with them. Once associate strongly with them the idea of danger, and that too of the most awful kind, and he will easily cease mental exertion on their approach. Studious men, when they are conscious of the feelings we have mentioned, often consider them as the effects of indolence, and endeavour to shake them off by increased exertion; but let them remember, that they thus endanger not only bodily health, but also the proper exercise of every useful faculty of mind. This weariness and sense of fatigue, is widely different from that sluggishness and unwillingness for exertion which attend the gross and sensual,—those whose aim is fixed on nothing great in attempt, or noble in accomplishment. Their fatigue is weariness before labour: like some domestic fowl, unwarily raised into air, which flaps its heavy pinions for a moment, and then drops to its accustomed level, they require constant incentive to keep them on the wing; while those to whom we recommend caution, are like some noble bird of heaven, stretching its flight towards ethereal regions, which soars and soars, unconscious of fatigue and reckless of danger, till it dies in the clouds.

Another means of preventing disease from over-exercise of mind, is, to have several objects of study, which, if they do not become main objects, may serve for amusement as well as instruction; for, as Crichton seems well to have observed, "when the objects of study are numerous, and do not belong to one subject only, the habit of easily passing from one association of ideas to another easily increases; and thus, all the faculties of the mind have a more equal degree of exercise." Change of employment, of either mind or body, relieves fatigue.

The studious man of genius should often indulge in social company: this tends to divert the mind from its austere pursuits, and breaks-in upon those strong associations, which else might lead to evil consequences. The sage, in Rasselas, says, "If I am accidentally left alone for a few hours, my inveterate persuasion rushes upon my soul, and my thoughts are chained down by an irresistible violence: but they are soon disentangled by the prince's conversation, and are instantaneously released at the entrance of Pekuah."

Another preventive means, is, never to engage in study just before retiring to rest; for, besides the corporeal effects produced by loss of sleep, from the associations thus excited continuing to act after the voluntary exertion of attention has ceased, these associations make a much deeper impression on the mind in the darkness and silence of night, than during the interruptions and variety of the day.

With the means enumerated must be used exercise of body, which preserves it as a machine in proper order to be acted on by the mind.

For the cure of the diseases of genius we refer to the writings of Rush, whose comprehensive mind was much applied to this subject; and gladly ought we to receive the instructions of such a man on any subject. The powers of his genius were so guided, as to be productive of lasting good to his country and to mankind. His enemies, whilst they prowl around his grave, and vainly endeavour to tear thence the laurel which will ever be green over it, still profit by his labours. When the ephemeral fame of such men is withered, and their names are repeated by no tongue, Rush must be remembered as one, than whom the medical profession can boast none greater—none better!*

* We shall be thankful to Graduates who will send us copies of their inaugural theses. We are disposed to publish select ones, or extracts from them, in our Journal occasionally.—EDIT.

